# Determining Cluster-Specific Differences in the Number of Days Required to Reliably Predict Habitual Physical Activity: Intraclass Correlation Resampling Analysis

**DOI:** 10.2196/64323

**Published:** 2026-02-10

**Authors:** Conor Jordan Murphy, Gabriel M Jouan, Katrin Y Friðgeirsdóttir, Anna Sigridur Islind, Jose M Saavedra, María Óskarsdóttir, Erna Sif Arnardóttir

**Affiliations:** 1 School of Technology, Reykjavik University Sleep Institute Reykjavík University Reykjavik, Capital Region Iceland; 2 Sports Science Department, Physical Activity, Physical Education, Sport and Health (PAPESH) Research Centre School of Social Sciences Reykjavík University Reykjavik, Capital Region Iceland; 3 Department of Computer Science Reykjavík University Reykjavik, Capital Region Iceland

**Keywords:** physical activity, accelerometry, reliability, intraclass correlation coefficient, human behavior, data, wearable devices, wearables, self-reporting, habitual, exercise, step counts, steps, walking

## Abstract

**Background:**

Previous research has attempted to determine the minimum number of days of accelerometry required to reliably reflect an individual’s physical activity. However, human behaviors on a day-to-day basis can be highly variable. As a consequence, the number of days required to reliably predict habitual physical activity is dependent on the variability that exists within an individual. There is a concern that adopting generic recommendations from previous research could provide unreliable estimates by failing to represent individuals with specific physical activity patterns.

**Objective:**

The main aim of this study was to identify clusters of individuals with distinct physical activity patterns and to determine if the number of days of accelerometry data required to reliably estimate short- (7 days) and medium-term (28 days) physical activity differed between each unique cluster.

**Methods:**

Accelerometry data were retrieved from 2 independent research studies. Participants during each study had their physical activity recorded using a Withings Scanwatch (Withings Health Solutions). Following a data eligibility process, agglomerative hierarchical clustering was used to identify clusters of individuals based on their physical activity. The clusters were determined using 4 dimensions; mean, SD, skewness, and kurtosis of the step count data. Intraclass correlation coefficients (ICCs) of step count were then calculated within each physical activity cluster. A series of ICCs were computed by separately comparing the average step count across the full periods (7 and 28, for the short- and medium-term analysis, respectively) to a series of averaged subsamples (ranging from 1-6 days and 1-27 days, for the short- and medium-term analysis, respectively). For each subsample, 500 random combinations were generated and compared, providing a distribution of ICCs for each subsample. An ICC of ≥0.80 identified when the subsample of days was sufficient to achieve appropriate reliability.

**Results:**

Of 258 participant datasets, 149 were eligible for the short-term analysis and 64 were eligible for the medium-term analysis. Following agglomerative hierarchical clustering, 4 and 3 clusters of sufficient size (n≥12) were identified in the short-term and medium-term analyses, respectively. When considering the short-term analysis, to achieve a mean ICC score greater than or equal to 0.80, using all randomized combinations, the number of days ranged from 2 to 6 days depending on the physical activity cluster. For the medium-term analysis, the number of days required to achieve a mean ICC score greater than or equal to 0.80 ranged from 6 to 11 days. The short-term analysis clusters displayed more diversity in physical activity patterns than the medium-term analysis.

**Conclusions:**

Physical activity patterns influence the number of days required to estimate habitual physical activity. Thus, to avoid unreliable estimates of physical activity, which could significantly impact the interpretation of results, researchers should be mindful of the physical activity patterns of their sample before adopting generic recommendations.

## Introduction

Accelerometers are wearable devices that measure the acceleration of the body region to which the device is secured. Commercially, the signals are then processed and transformed into physical activity metrics that are relatable to the consumer, such as daily step count. The popularity of accelerometry as a method to quantify physical activity is growing due to the objectivity and availability of such devices. Importantly, the use of accelerometry provides an alternative option to self-reporting questionnaires, which can lack strong reliability and validity [1].

The field is rapidly developing with the advancement of new technology and the commercialization of devices. As a consequence, the approaches used to interpret collected data can vary and lack a consensus [2]. An area of importance relates to the minimum number of valid days required to reliably reflect a person’s habitual level of physical activity [3]. Unfortunately, many studies with important implications, such as those linking physical activity to health outcomes, have used varying criteria to define a participant’s habitual level of physical activity. For example, the United Kingdom biobank guidelines advise researchers to exclude participants from their accelerometry dataset who have fewer than 3 valid days of data [4], which has been adopted in many impactful studies [5-7]. In contrast, the National Center for Health Statistics, which administers the National Health and Nutrition Examination Survey in the United States, does not advise on the number of valid days that should be available to validate inclusion when using their accelerometry datasets. As a consequence, researchers have adopted various inclusion criteria ranging from 1 to 5 days [8-10].

From a research perspective, the fewer days required could help relieve the study burden on the participant and better support the use of study resources. Past reviews have suggested 4 days would suffice to achieve sufficient reliability [2,11]. Furthermore, a more recent comprehensive study using a large Singaporean sample recommended that at least 3 and 5 measurement days of step count data were needed to predict weekly and monthly time windows, respectively [12]. Despite the existing recommendations, providing a consensus for the minimum number of days required to predict habitual physical activity can be challenging and fraught with error if the uniqueness of the sample in question is not considered. Human behaviors on a day-to-day basis can be highly variable; thus, the number of days required to reliably predict habitual physical activity is dependent on the variability that exists within an individual [13]. Intuitively, individuals with high consistency or limited variability in physical activity may be predicted with fewer days than an individual with low consistency and high variability. As a consequence, the identification of an optimal number of days could be problematic. Another source of consideration relates to the formula used to calculate reliability. In the physical activity literature, the intraclass correlation coefficient (ICC) is commonly used to grade the reliability of physical activity and a common standard used to indicate acceptable reliability is an ICC of 0.8 [13,14]. However, broadly, this formula approximates the within-subject variability by comparing its magnitude to the between-subject variability. Thus, an increase in diversity between individuals will inherently reduce the reliability score without any change in variability within individuals. This situation poses a potential concern that predictions from large heterogenic samples may not translate to groups of individuals with specific physical activity patterns.

Therefore, the main aim of this study was to identify clusters of individuals with distinct physical activity patterns and to determine if the number of days of accelerometry data required to reliably estimate short- (7 days) and medium-term (28 days) physical activity differed between each unique cluster. This study used clustering analysis to allocate individuals from a heterogenic population into distinct physical activity clusters. Subsequently, reliability estimates were derived for each cluster and compared. The hypothesis was that the number of days to reliably predict (1) short- and (2) medium-term physical activity differs between clusters.

## Methods

### Study Design

This is a secondary, longitudinal analysis using combined data from 2 independent studies. Only the objectively measured physical activity data (daily step counts) and demographic descriptors were used from each dataset. All other variables from the original studies were excluded from the current analysis. The analysis was conducted at Reykjavik University in Iceland as part of the European Union Horizon 2020-funded Sleep Revolution research project [15].

### Setting

Study 1 was an observational study conducted at the Reykjavik University Sleep Institute, aimed at exploring the associations between physical activity and markers of obstructive sleep apnea (OSA) severity [16]. A total of 66 adults were recruited, representing a broad range of sleepers, including healthy individuals, snorers, and those with suspected or diagnosed OSA. Each participant underwent a 3-night, self-applied somnography study at home, along with assessments of anthropometry, body composition, and both subjective and objective physical activity. Importantly, for the purpose of this analysis, objective physical activity was measured using a smartwatch device (Withings Scanwatch, Withings Health Solutions) worn on the nondominant wrist for a 3-month period.

Study 2 was a 12-week, 3-arm randomized controlled trial conducted at the Reykjavik University Sleep Institute [17]. The study was registered in ISRCTN 16974764*.* It aimed to evaluate the effects of an exercise program and a lifestyle app on OSA, physical health, and quality of life in adults with mild-to-moderate OSA or habitual snoring. A total of 192 eligible participants were randomized into exercise, app, or control groups. Randomization was stratified using an algorithm to ensure balance across age, gender, BMI, and apnea-hypopnea index (AHI). Participants completed baseline assessments, including a one-night type 3 sleep study, body composition, and physical activity measures. The exercise group attended structured sessions 3 times per week, and the app group engaged in daily behavioral tasks via a health app (Sidekick Health, Reykjavík). Objective physical activity was assessed via a smartwatch device (Withings Scanwatch, Withings Health Solutions) worn on the nondominant wrist over the 12-week study period.

### Participants

Participants from each study, who are described in the subsequent paragraphs, were pooled to explore habitual physical activity patterns. Study 1 recruited a heterogeneous sample from the general adult population (≥18 years) with the goal of reflecting natural variation in age, sex, and body morphology. Participants were not excluded based on sleep health status. Study 2 was a randomized controlled trial targeting adults aged 18–50 years of age that were categorized as overweight or obese (BMI ≥25<42 kg/m²), who were physically inactive and had either mild-to-moderate OSA (AHI ≥5 and <30) or habitual snoring (≥10%). Shift workers and individuals undergoing OSA treatment were excluded. Eligible participants underwent a one-night type 3 sleep study to confirm OSA.

### Variables

The primary variable analyzed was daily step count, measured objectively using accelerometry-based methodology. Step count served as the key indicator of physical activity throughout the study period.

### Data Measurements

#### Demographics

Data were collected using a custom-developed questionnaire. REDCap (Research Electronic Data Capture) survey software version 9.3.1 (Vanderbilt University) was used to collect participant responses [18,19].

#### BMI

Height and weight (digital scale; TANITA MC-780, Tanita Corporation) were measured and used to calculate BMI.

#### Smartwatch

Participants were instructed to wear a smartwatch (Withings Scanwatch, Withings Health Solutions) for the study period. The smartwatch generated physiological and physical activity parameters that included step count, distance covered, elevation, sleep, heart rate, oxygen saturation, passive calories, and active calories. Due to the substantial correlations between the physical activity parameters, step count was selected as the parameter of primary interest. The generated data were available in two forms: (1) timestamped data and (2) daily aggregated data. Additional details of the smartwatch are described by [20].

#### Bias

Several potential sources of bias were considered in the study design and analysis. First, selection bias was likely present in Study 2, as participants were specifically recruited based on specific inclusion criteria around OSA. However, Study 1 reflected a broader segment of the general adult population and may have helped offset this limitation by introducing more heterogeneity in age, sex, BMI, and physical activity profiles. Nonetheless, the combined sample remains somewhat weighted toward individuals with OSA. Measurement bias was addressed by using objective step count data from smartwatches rather than self-reported physical activity, reducing recall and reporting biases. However, inaccuracies could arise from nonwear time or device limitations; to mitigate this, systematic methods were used to exclude days with insufficient wear time [2,21]. Similarly, to address potential bias from missing days, nonconsecutive valid days were allowed but only within specified windows described in more detail in the Sample Size section.

### Sample Size

All participants from Study 1 and Study 2 were considered eligible for analysis, but their inclusion depended on passing specific wear-time criteria.

#### Valid Day

Given the longitudinal nature of the study, participants were not required to report nonwear time, which influenced the accuracy of step count data. Therefore, a systematic approach to identify nonwear periods was established. Gaps in the timestamped “calories earned” data exceeding 60 minutes were classified as nonwear periods. Summing the nonwear periods that occurred between consecutive sleep periods permitted the calculation of wear time during waking hours. In line with previous approaches and retaining statistical power [2,21], days with less than 10 hours of wear time during waking hours were considered insufficient to accurately estimate step count and excluded.

#### Valid Period

Using the step count data filtered for valid days, one period of 7 days and another of 28 days were selected for each participant in the short- and medium-term analyses, respectively. Periods with nonconsecutive days were allowed if there were (1) 7 valid days within a 10-day period in the short-term analysis or (2) 28 valid days within a 40-day period in the medium-term analysis. Periods with full consecutive sequences or with the fewest days skipped were preferentially chosen, but if more than one of these sequences existed, then the selection was randomized. Nonconsecutive days were permitted due to the difficulty of achieving full consecutive sequences of data, particularly when considering the medium-term analysis. For the inspection of data, the chosen periods of 10 and 40 days were chosen to allow us to retain a sufficient sample size without compromising the representation of physical activity for the given period.

### Data Analysis of Quantitative Variables

**Figure 1:**
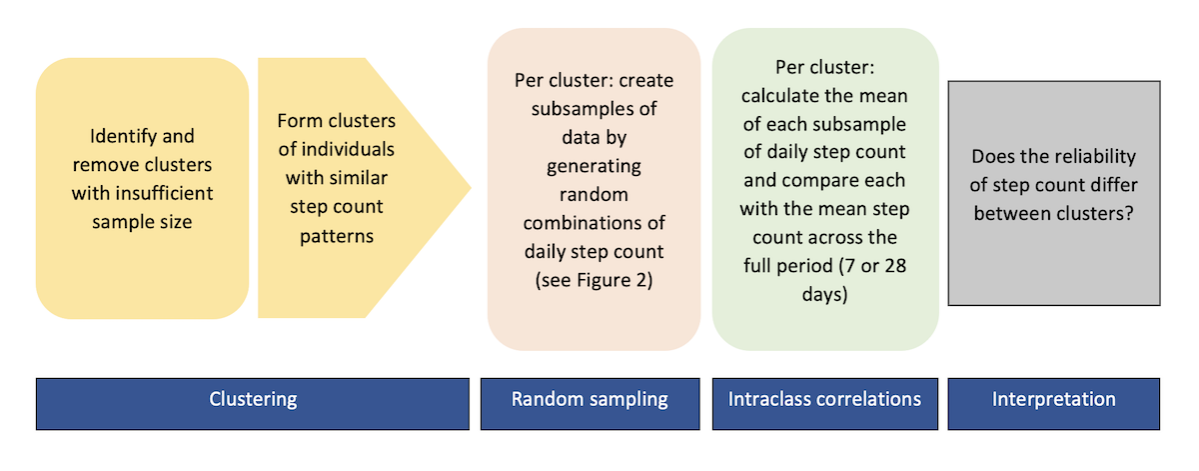
Methodological approach to study analysis.

The methodological approach to the analysis of the daily step count variable is displayed in a visual format in Figure 1. Using unsupervised machine learning, we identified clusters of individuals that shared more similar physical activity patterns than those in the other identified clusters. Agglomerative hierarchical clustering with complete-linkage, a distance-based algorithm, was used to identify clusters [22]. This form of clustering was chosen because it can be used to identify outliers and does not require a predetermined number of clusters. The clustering analysis was conducted for both short- and medium-term physical activity patterns, separately. The clustering analysis first involved running the algorithm to identify sufficiently sized clusters. Given that the calculated reliability metrics are influenced by between-subject variability, smaller clusters may be more prone to an underestimation or overestimation of reliability depending on participant similarities. Thus, if any clusters with less than 12 participants were identified, they were removed, and the remaining clusters were retained for the full analysis. The clusters were determined based on their similarities across 4 dimensions, those being the scaled and centered mean, SD, skewness, and kurtosis of the step count data. These dimensions represent the 4 moments of distribution, which quantitatively reflect each participant’s physical activity patterns in the context of an average, day-to-day variability and range of values.

### Statistical Analysis

**Figure 2:**
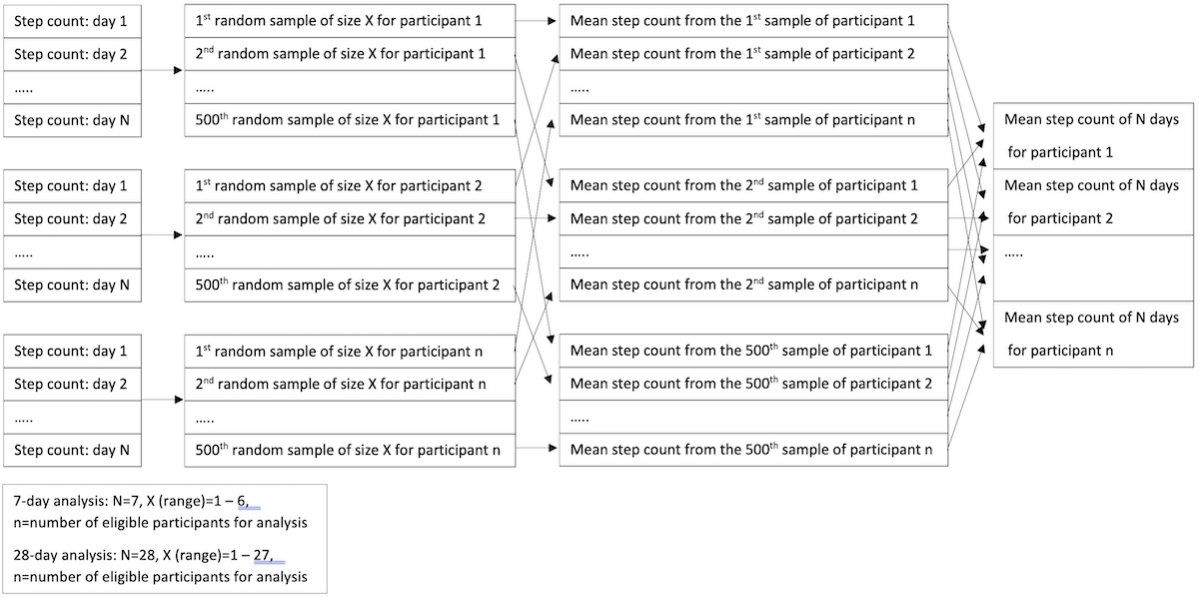
Data randomization of subsamples.

Following the identification of clusters, the reliability of step count within each cluster was determined, along with a baseline comparison (unclustered data). ICCs were derived using linear mixed models with one-way random effects. A series of ICCs were computed by separately comparing the average step count across the full period (7 and 28 days for the short- and medium-term analysis, respectively) to a series of averaged subsamples (ranging from 1 to 6 days and 1 to 27 days for short- and medium-term analysis, respectively). As the subsamples could include any combination of days from the full period, for each participant, resampling methods without replacement were used to ensure an appropriate number of combinations were tested. For each subsample, 500 random combinations were generated and compared to the average step count across the full period (see Figure 2). Prior testing of the data showed that when 500 combinations were used for ICC predictions, the results remained stable when repeated. Thus, the range of ICC values possible for each subsample comparison was produced. In line with previous research, a threshold value of 0.80 was used to infer “acceptable” reliability [13,14].

It is necessary to point out the benefits of the Monte Carlo resampling approach used. This approach reduces the influence of nonnormality and heterogeneity of within-subject variances by using aggregated participant-level means and repeated ICC estimations. Given that ICCs were computed from mean step counts, assumptions of normality and homogeneity of variance are less probable, as justified by the Central Limit Theorem. Additionally, using 500 iterations per subset further enhances the robustness of the reliability estimates by reducing the influence of a typical combinations.

All the analyses were conducted in R (version 4.3.2; R Core Team). Specifically, the “ICC” package was used to extract the ICCs.

### Ethical Considerations

Both studies received ethical approval from the National Bioethics Committee of Iceland and the Icelandic Data Protection Agency (21-170; 22-082). Participants provided written informed consent to participate, which also extended consent to secondary analysis. All data were stored anonymously and processed on a secure high-performance computing cluster to ensure efficient handling while maintaining data security and integrity. Finally, participants did not receive any compensation for their participation.

## Results

### Participants and Descriptive Data

Of the 258 original participants available from Study 1 and Study 2, a total of 149 participants possessed 7 valid days (>10 hour wear time during wake) across a 10-day period and were included in the short-term analysis, and 64 participants possessed 28 valid days across a 40-day period and were included in the medium-term analysis.

In the short-term analysis, 76 of the participants were male, and 73 were female. On average, the participants were 41 (SD 5) years of age and were categorized in the obese category (BMI: mean 31, SD 4). Age ranged from 19 to 76 years old, while BMI ranged from 19 to 42. In the medium-term analysis, half of the participants were male (n=37) and half of the participants were female (n=37). On average, the participants were 45 (SD 9) years of age and were categorized in the obese category (BMI: mean 31, SD 5). Age ranged from 23 to 65 years old, while BMI ranged from 20 to 41. The participants that were eligible for both analyses did not have any missing data.

### Outcome Data

#### Clustering in Short-Term Analysis

Following agglomerative hierarchical clustering, 4 clusters of sufficient size (n≥12) were identified from a visual inspection of the dendrogram. The clusters were chosen by attempting to achieve a balance between appropriate sample sizes and within-cluster cohesion. The 4 dimensions of daily step count used to form each cluster are reported in Table 1.

In comparison to the other clusters, Cluster 1 displayed moderate physical activity levels and day-to-day variability. However, Cluster 1 had a highly skewed physical activity pattern and high kurtosis, suggesting these participants tended to have days that, when compared to their habitual physical activity levels, were considered extremely low or high. In contrast, the other clusters had kurtosis values below or slightly above 3, suggesting the number of days classed as outliers (extreme high or low physical activity) was similar to or less than that seen in a normal distribution pattern. Cluster 2 displayed the lowest relative variability in physical activity levels, while participants in Cluster 3 had the lowest overall physical activity levels, with habitual levels of 4391 (SD 1058) steps per day. Cluster 4 displayed the highest physical activity levels (mean 7179, SD 1028 steps per day), but also the highest day-to-day variability. Given the low kurtosis and moderate skewness, this would suggest the participants in Cluster 4 displayed a mixture of physical activity days.

**Table 1:** Dimensions of daily step count used to isolate clusters of individuals in the short-term analysis.

Short-term Cluster (n)	Mean steps, mean (SD)	SD steps, mean (SD)	Skewness, mean (SD)	Kurtosis, mean (SD)
1 (n=36)	5883 (1298)	2192 (727)	1.28 (0.48)	5.34 (1.08)
2 (n=22)	5980 (1538)	1619 (685)	0.82 (0.26)	3.67 (0.99)
3 (n=42)	4391 (1058)	1467 (586)	0.50 (0.35)	2.20 (0.83)
4 (n=30)	7179 (1028)	3068 (1132)	0.55 (0.37)	1.96 (0.70)

#### Clustering in Medium-Term Analysis

Following agglomerative hierarchical clustering, three clusters of sufficient size (n≥12) were retained by visually inspecting the dendrogram. The four dimensions of daily step count used to form each cluster are reported in Table 2.

Compared to the short-term analysis, the clusters within the medium-term analysis displayed less diversity. Modest day-to-day variability in physical activity was found in Cluster 1, but the participants in this cluster had the highest physical activity levels (mean 7671, SD 1052 steps per day). In contrast, participants in Cluster 2 displayed the lowest levels of physical activity (mean 4746, SD 947 steps per day) with the highest relative variability in physical activity. Cluster 3 displayed the least relative variability in physical activity and a low degree of skewness. This suggests that the physical activity levels for these participants were usually around the mean alongside an expected number of higher and lower physical activity days (normal distribution). The number of extremes in physical activity was small in all clusters and did not display distinctly different tails to a normal distribution pattern.

**Table 2:** Dimensions of daily step count used to isolate clusters of individuals in the medium-term analysis.

Medium-term Cluster (n)	Mean steps, mean (SD)	SD steps, mean (SD)	Skewness, mean (SD)	Kurtosis, mean (SD)
1 (n=23)	7671 (1052)	2955 (655)	0.66 (0.45)	3.65 (1.11)
2 (n=14)	4746 (947)	2265 (381)	0.87 (0.24)	3.31 (0.58)
3 (n=16)	5775 (988)	1764 (398)	0.31 (0.28)	2.87 (0.72)

### Main Results

#### Intraclass Correlations in Short-Term Analysis

**Figure 3:**
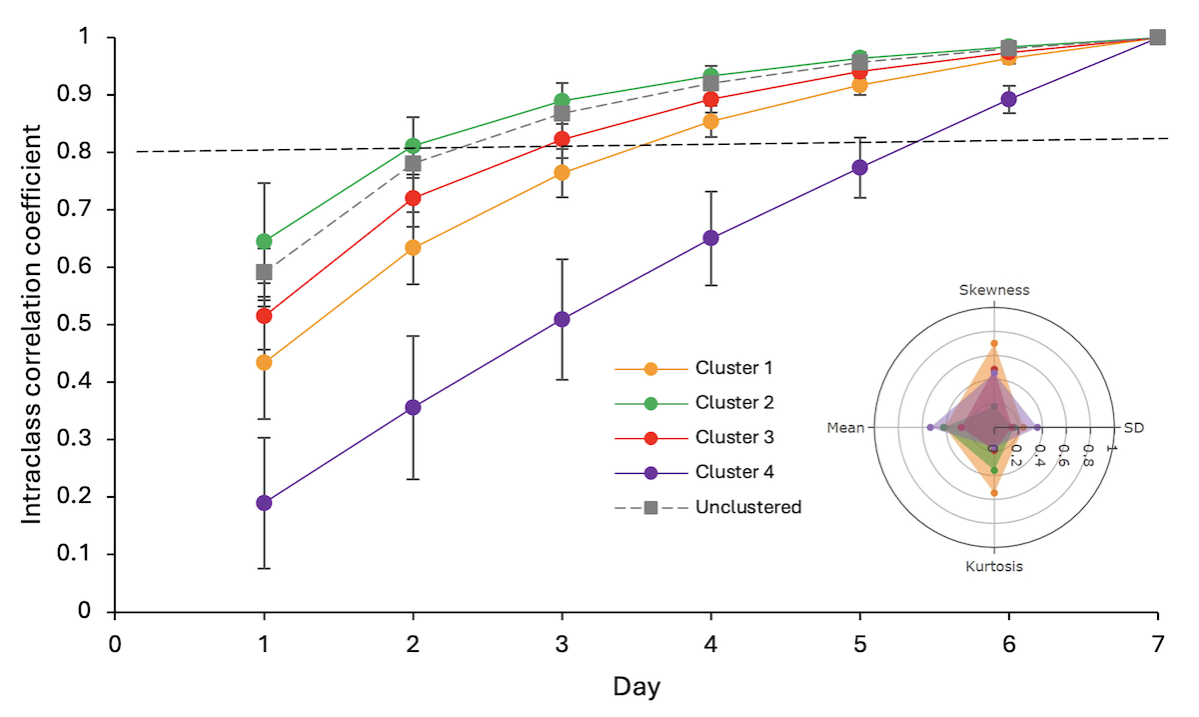
Range of intraclass correlation coefficients across all randomized combinations for each cluster from short-term analysis. Scores ≥ 0.80 are classed as “acceptable” agreement. Data are mean (SD).

To achieve a mean ICC score above 0.80, calculated using all randomized combinations for each day subset, Cluster 2 required 2 days, Cluster 3 required 3 days, Cluster 1 required 4 days, and Cluster 4 required 6 days (Figure 3). The minimum number of days needed to achieve an ICC score above 0.80 in all 500 random combinations was 4 in Clusters 2 and 3, 5 in Cluster 1, and 7 in Cluster 4. As a baseline comparison, when the data were unclustered, 3 days were required to achieve a mean ICC score above 0.80 in all 500 random combinations.

#### Intraclass Correlations in Medium-Term Analysis

**Figure 4:**
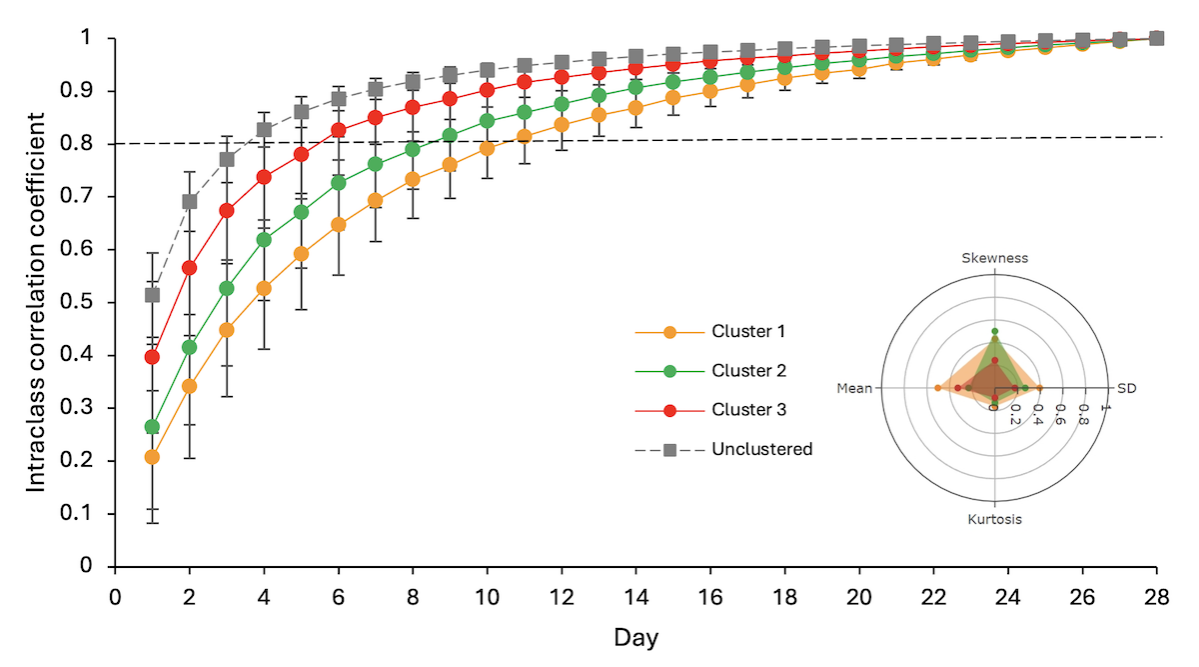
Range of intraclass correlation coefficients across all randomized combinations for each cluster from medium-term analysis. Scores ≥ 0.80 are classed as “acceptable” agreement. Data are mean (SD).

In order to achieve a mean ICC score greater than 0.80, calculated using all randomized combinations for each day subset, Cluster 3 required 6 days, Cluster 2 required 9 days, and Cluster 1 required 11 days (Figure 4). The minimum number of days needed to achieve an ICC score above 0.80 in all 500 random combinations was 11 in Cluster 3, 18 in Cluster 2, and 17 in Cluster 1. As a baseline comparison, when the data were unclustered, 4 days were required to achieve a mean ICC score above 0.80 in all 500 random combinations.

## Discussion

### Principal Findings

The main aim of this study was to identify clusters of individuals with distinct physical activity patterns and to determine if the number of days of accelerometry data required to reliably estimate short- (7 days) and medium-term (28 days) physical activity differed between each unique cluster. Clustering analysis confirmed that, in a heterogenic sample, clusters of individuals could be identified based on their physical activity patterns. In agreement with our hypothesis, the physical activity patterns of different individuals were highly relevant to the number of days needed to reliably predict both short- (7 days) and medium-term (28 days) physical activity, ranging from 2 to 6 days and 6 to 11 days, respectively, by cluster. Therefore, the minimum number of days should be assessed within a particular study cohort, instead of relying on generic recommendations.

One major consideration of using accelerometry for longitudinal physical activity research is that participant monitoring is a continuous process, which can require significant study resources and can challenge participant adherence. Therefore, having the flexibility to monitor less or lose more days, without compromising the validity of the data, is of significant benefit. However, the results from the current study show that, while this is possible, it can be erroneous to use a generic number of days across all participants. This supports the need to be cautious when considering a one-size-fits-all approach when it comes to collecting accelerometry data. For example, past reviews have suggested 4 days would suffice to achieve sufficient reliability in physical activity [2,11]. However, based on the results from the current study, this will be highly dependent on the physical activity patterns that exist within the specific research sample being studied. Another potential limitation of previous recommendations is the use of the Spearman-Brown prophecy formula to estimate reliability [23-25]. This statistical method depends on achieving homogeneity of variances, which may be unrealistic to achieve in many datasets. The resampling approach in this study helped address this problem, as it does not rely on this formula and is less sensitive to violations of homogeneity of variance. By repeatedly calculating ICCs from randomly selected combinations of days, the method reduces the influence of unequal within-subject variances on individual ICC estimates, resulting in a more robust overall estimate of reliability. Similarly, a nonclustered ICC may violate the assumption of homogeneity of variance if the physical activity patterns of the sample are diverse. Clustering can help group individuals with similar within-subject variance, improving the likelihood that model assumptions are satisfied.

The results from both the short- and medium-term analyses highlighted that clusters with relatively low variability in their physical activity required fewer days to reliably estimate physical activity. In contrast, clusters with high variability required significantly more days. Referring specifically to the data, depending on the cluster, the mean number of days required ranged from 2 to 6 for the short-term analysis and 6 to 11 for the medium-term analysis. This makes generalized recommendations as reported in previous research [26-28] difficult to provide. Interestingly, within this study’s sample, the physical activity patterns of clusters in the medium-term analysis were more similar to each other than those observed in the short-term analysis. This implies reduced variability as participants settle into comparable daily activity patterns with larger measurement windows. Nonetheless, it is important to mention that, for one cluster, the minimum number of days needed to achieve sufficient reliability in all 500 random combinations was still noteworthy (17 days).

To an extent, these results agree with the work by [12] that recommended at least 3 and 5 measurements days of step count data were needed to predict weekly and monthly time windows, respectively. However, it highlights the risk of adopting the minimum limit of these recommendations for all participants. Instead, an approach that first attempts to understand the sample that is being studied could be beneficial. For example, if pilot testing is being conducted, this could provide an opportunity to collect prior accelerometer data on the group of participants. Depending on whether the physical activity patterns are expected to be similar or not, a cluster analysis could be used to determine the minimum number of days needed for each subset of participants. Alternatively, while physical activity questionnaires may provide an inexpensive and less-demanding method to group individuals, they can be prone to recall bias, as we have previously shown [16].

Practically, this research demonstrated that a heterogeneous sample can be divided into subgroups with distinct physical activity patterns. As a consequence, these clusters can influence the reliability of predicting habitual physical activity and, thus, the broader interpretation of the results. Previous research linking physical activity to health outcomes has estimated a participant’s habitual level of physical activity using subsamples ranging from 1 to 5 days [8-10]. Unfortunately, the validity of such results is tied to the accuracy of this physical activity estimation. Researchers who do not fully consider the potential heterogeneity in their sample may be at potential risk of underestimating within-subject variability, which could potentially influence their interpretation of the results. Therefore, safeguards should be considered to improve physical activity estimates that include clustering, using appropriate ICC methods, and including complementary reliability metrics (standard error of measurement, coefficient of variation, etc).

Another point of consideration is that, in research studies, accelerometry is often used to quantify the rest-activity cycle of participants, which spans physical activity, sedentary time, and sleep. While this research focused on physical activity, the reliability of sedentary time and sleep will also display different levels of within-subject variability. Therefore, if researchers intend to quantify all 3 components of the rest-activity cycle, it is important to consider whether the number of days chosen is sufficient to capture the variability across all 3 components. For example, before individual sleep behaviors become stable, they require many more days of data than the general recommendation of 14 days [3]. However, more research exploring clustered samples is required because results from grouped data have reported differing results [29-31], which is likely a reflection of the level of heterogeneity across the sample in each analysis.

The current study is not without its limitations. First, the specific results of the current study reflect the discrete physical activity patterns of the analyzed sample. Nonetheless, the core concepts are universal and generalizable to other research samples in this area. With a larger sample size, more clusters may have been identified, or rather, a greater number of participants may have been placed within each identified cluster. For example, a number of clusters that were identified had insufficient sample sizes to conduct the analysis on and thus were excluded. It is plausible that the individuals with these excluded physical activity patterns may have required even more or fewer days to reliably estimate their physical activity over time, compared to those seen in the reported results. Importantly, while the physical activity patterns of the excluded individuals were uncommon in this study, they may better reflect physical activity patterns seen in a different study. Notably, while our sample was relatively heterogeneous, there was a higher percentage of individuals with large BMIs, which may have contributed to a small bias, as BMI has been shown to be associated with lower physical activity patterns [32]. Nonetheless, if the sample in this study had more variance, it likely would have strengthened the results while also improving the study’s generalizability. In the current study, 10 or more hours of wear time during waking hours for a given day was considered sufficient for inclusion. Therefore, certain days with missing physical activity data will inherently be included and can influence reliability predictions [24]. Nonetheless, this decision was made to align with previous approaches [2,21], and to balance accuracy with the retention of information. Finally, ICCs are based on the proportion of between-to within-subject variance; thus, results from separate samples can differ based on within-subject variance (day-to-day variability of steps within a participant) but also, between-subject variance (variability of steps between participants). As alluded to in the introduction, this poses a potential concern that recommendations from large heterogeneous samples may misrepresent research samples that are more homogenous in nature. Unfortunately, while widely used, this is the limitation of using ICCs in the context of estimating physical activity. Importantly, our clustering analysis attempted to limit the between-subject variance by identifying clusters of individuals with similar physical activity patterns based on the 4 moments of distribution.

### Conclusion

In conclusion, the number of days required to reliably estimate physical activity differs between clusters of individuals characterized by distinct physical activity patterns across both the short- and medium-term. To avoid unreliable estimates of physical activity, researchers should be mindful of the sample they are studying and how this may influence the minimum number of days required to reliably reflect physical activity. This study showcases that one-size, indeed, does not fit all when it comes to collecting accelerometry data.

## Abbreviations

AHI: apnea-hypopnea index
ICC: intraclass correlation coefficient
OSA: obstructive sleep apnea
REDCap: Research Electronic Data Capture
